# Theophylline Toxicity: A Differential to Consider in Patients on Long-Term Theophylline Presenting With Nonspecific Symptoms

**DOI:** 10.7759/cureus.48480

**Published:** 2023-11-08

**Authors:** Jing W Goh, Myat M Thaw, Jasim U Ramim, Rahul Mukherjee

**Affiliations:** 1 Respiratory Medicine, Heartlands Hospital, Birmingham, GBR

**Keywords:** therapeutic window, therapeutic drug monitoring, chronic obstructive pulmonary disease(copd), theophylline poisoning, theophylline

## Abstract

Theophylline has been used for decades as a bronchodilator to treat asthma and chronic obstructive pulmonary disease (COPD). The precise mode of action is still uncertain. Since beta-2 agonists are at least as effective as theophylline as a bronchodilator while having fewer side effects, theophylline has become less popular in recent clinical practice as the first-line treatment in patients with airway obstruction due to its narrow therapeutic window, which necessitates frequent level monitoring, severe side effects that can be fatal, and interactions with other medications. Patients with a chronic theophylline overdose often present with nonspecific gastrointestinal symptoms, which can result in misdiagnosis for a variety of gastrointestinal conditions. Convulsions that may be fatal can occur as a result of a theophylline overdose. Therefore, it is important to manage individuals who have been taking theophylline for a long period and have nonspecific cardiac or gastrointestinal symptoms with a high index of suspicion for theophylline toxicity. We present a case of a COPD patient who had no regular theophylline level monitoring for two years. He presented with vague gastrointestinal symptoms for the past six months and was initially suspected of having gastrointestinal cancer due to weight loss caused by decreased oral intake due to nausea and vomiting. He was suspected of having theophylline toxicity because he was on long-term theophylline and did not have frequent theophylline level monitoring. His theophylline level was measured and found to be high at 28.9 mL/L (normal level, 15 mL/L). He was given conservative treatment, including intravenous fluid and electrolyte replacements, and close monitoring of his theophylline levels.

## Introduction

Theophylline is a potent methylxanthine that has been used as a bronchodilator in clinical practice for decades to treat individuals with airway obstructions such as asthma and chronic obstructive pulmonary disease (COPD) due to its bronchodilation and mild anti-inflammatory effects. Although the molecular mechanism of action of theophylline is unknown, it has been shown that it demonstrates bronchodilation effects by reducing adenosine actions, which subsequently reduces histamine synthesis known to cause bronchospasm. It also inhibits phosphodiesterase (PDE), which increases cyclic adenosine monophosphate (cAMP) and, as a result, relaxes airway smooth muscles. Theophylline also exposes one to a wide range of adverse consequences as a result of the aforementioned molecular processes, from nonspecific symptoms like nausea, vomiting, and headaches to potentially lethal cardiac arrhythmias and seizures. These side effects are generally dose dependent, with larger theophylline doses resulting in theophylline toxicity [1].

Theophylline has a narrow therapeutic window, with a therapeutic plasma level of 10-15 mg/L [2]. Although theophylline toxicity is more common when plasma levels exceed 20 mg/L, severe symptoms may occur even when plasma levels are within the normal therapeutic range [1,3]. Theophylline dose required to achieve therapeutic concentration varies from person to person and is primarily regulated by theophylline hepatic metabolism in the liver by the CYP1A2 and CYP2E1 enzymes [1]. Drugs that interfere with theophylline hepatic clearance predispose patients to adverse effects. Furthermore, these side effects are nonspecific and usually go undiagnosed in clinical practice, resulting in chronic toxicity. Individualization of theophylline dosage and regular monitoring of theophylline levels are therefore crucial in people on theophylline.

## Case presentation

A 69-year-old man with COPD, paroxysmal atrial fibrillation, hypertension, and osteoarthritis arrived at the emergency department after experiencing two weeks of intermittent vomiting. For the past six months, he had been experiencing sporadic nausea and dyspepsia. He visited his primary care physician two months before his hospitalization for nausea and dyspepsia and was recommended for an oesophageal-gastro-duodenoscopy (OGD) to rule out upper gastrointestinal malignancy since he had lost weight due to poor oral intake. His OGD eventually came back as normal. He did not have fever, palpitation, dyspnea, tremor, headache, confusion, or seizure.

He was diagnosed with emphysema based on chest computed tomography (CT) findings 12 years ago and began using Trimbow inhalers for COPD. He was a smoker with a 50-60 pack-year history who had quit two months ago. After six hospitalizations for COPD exacerbation in a year, he was eventually started on slow-released theophylline 200 mg tablets twice a day for control. He has had no further COPD exacerbations in the four years since starting theophylline.

On presentation, the patient was alert and not confused or agitated. His heart rate was 108 beats per minute, and his blood pressure was 94/52 mmHg. The rest of the vital signs were in the usual range. His physical examination revealed no abnormalities except rapid cardiac rate. His admission blood tests revealed hypokalaemia, hypomagnesaemia, hypocalcaemia, hypophosphatemia, and hypoalbuminemia (Table 1). A recent thyroid function test was normal. His ECG on hospital admission showed sinus tachycardia with a prolonged QT interval >500 milliseconds.

**Table 1:** Laboratory results at admission ALT: alanine transaminase, ALP: alkaline phosphatase, CCA: corrected calcium, INR: international normalized ratio, eGFR: estimated glomerular filtration rate, Na+: sodium, K+: potassium, Mg+: magnesium, PO4+: phosphate

Labs (unit)	Normal range	Value
Plasma		
Haemoglobin	120-160	113
Mean cell volume	78-98 FL	103.9
White cell count	4-11 x 10^9^/L	11.05
Platelets	140-400 x 10^9^/L	364
INR	-	2.1
Na+	133–146 mmol/L	138 mmol/L
K+	3.5–5.3 mmol/L	2.6 mmol/L
Urea	133–146 mmol/L	138 mmol/L
Creatinine	2.5–7.8 mmol/L	4.3 mmol/L
eGFR	59–104 μmol/L	85
Mg++	0.7–1.0 mmol/L	0.53 mmol/L
PO4+	0.74–1.4 mmol/L	0.38 mmol/L
CCA	2.2-2.6 mmol/L	1.94 mmol/L
Albumin	35–50 g/L	18 g/L
ALT	<41 U/L	22 U/L
ALP	30-130 U/L	196 U/L
Bilirubin	<21 μmol/L	08 μmol/L

Outcome and follow-up

Given the absence of routine theophylline level monitoring over the previous two years, theophylline toxicity was considered to be the cause of generalized gastrointestinal symptoms. While waiting for the theophylline level results, theophylline was discontinued. His theophylline level was 16.4 mg/L two years ago before this current admission.

He was diagnosed with theophylline toxicity after his theophylline level was found to be 28.9 mg/L on the second day of hospital admission. He was subsequently transferred to a respiratory ward and had his heart rate monitored. A low dose of bisoprolol was administered to control the heart rate together with intravenous fluids with potassium, magnesium, phosphate and calcium replacement. His heart rate was under control the following day and electrolyte imbalance was restored after two days of intravenous electrolyte replenishment. Therefore, bisoprolol was continued, and his daily theophylline levels were checked, which demonstrated that his theophylline levels steadily improved from 28.9 to 12.8 mg/L after two days of conservative treatment. His gastrointestinal symptoms had subsided after five days in the hospital. Although his theophylline level came back down within the therapeutic window and eventually to the sub-therapeutic level (<2 mg/L) on the sixth day of admission, it was never re-commenced. Later, while his hospital discharge was being evaluated, he contracted COVID-19 infection and died from secondary pneumonia. Independent death adjudication by two medical examiners ascertained that the death was not due to theophylline toxicity.

## Discussion

Theophylline has a narrow or small therapeutic index (TI). TI is the ratio that compares the blood concentration at which a drug becomes toxic and the concentration at which the drug is effective. The larger the TI, the safer the drug. If the TI is small (the difference between the two concentrations is very small), the drug must be dosed carefully and the person receiving the drug should be monitored closely for any signs of drug toxicity. This and its interaction with other medicines restricts its usage as first-line treatment for asthma and COPD. In comparison to nebulized ß2-agonists, studies have found no advantage to using theophylline in the treatment of patients with acute asthma. As a result, it has been reserved for patients who do not respond to ß-agonists [1]. Our patient had persistent gastrointestinal symptoms that were nonspecific and easily misdiagnosed as other gastrointestinal disorders such as peptic ulcer, gastro-oesophageal reflux disease, constipation, irritable bowel syndrome, gastrointestinal cancer and so on. Given that theophylline toxicity symptoms are frequently nonspecific, it is not surprising that the above-mentioned differential diagnosis was suspected in our instance. Our patient's symptoms, which included cardiac arrhythmias, nausea, vomiting, and dyspepsia, were induced by theophylline's phosphodiesterase inhibition and adenosine antagonistic actions [1]. Furthermore, our patient experienced electrolyte issues such as hypomagnesaemia and hypokalaemia, which were most likely caused by theophylline-induced high catecholamine levels [4]. Given that he had sinus tachycardia at the time of presentation, undetected theophylline intoxication would have resulted in death. Therefore, a high level of suspicion for theophylline toxicity is necessary for prompt diagnosis and treatment in individuals taking regular theophylline and experiencing nonspecific gastrointestinal symptoms.

This case demonstrated typical theophylline toxicity in a person who did not undergo routine theophylline level monitoring. Regular serum monitoring is required to avoid theophylline toxicity, which can be fatal due to its narrow TI. Theophylline is metabolized in the liver before being excreted via the kidneys. Medication that interferes with the cytochrome P450 enzymes may lead to theophylline toxicity. In addition, comorbidities including demographic and socioeconomic factors may potentially affect the drug's clearance rate, raising the theophylline level [5]. Theophylline monitoring should be done once a year for well-managed adults and once every six months for children when given orally. If the loading dose was given intravenously, the level must be monitored 30 minutes after the therapy. If the medicine was provided via infusion, it should be monitored for 12 to 24 hours after the initial infusion and for another 24 hours after the second infusion [6]. In this case, the patient received theophylline as part of his COPD therapy from a respiratory clinician and was unaware of the importance of regular theophylline level monitoring, resulting in his late presentation to hospital. Despite the fact that he was not taking any medications that could interact with theophylline and cause toxicity, his theophylline level remained high. This indicates that theophylline levels should be monitored on a frequent basis to avoid such incidents.

Theophylline toxicity is treated based on clinical symptoms and blood theophylline levels. Furthermore, it is influenced by a variety of factors, such as the amount consumed, the chronicity of the toxicity, and the individual's metabolic rate [7]. If the theophylline level is less than 60 mg/L, a single dose of activated charcoal can be administered. If the level is more than 60 mg/L, repeated oral doses of activated charcoal can be used to treat the condition up to the appropriate range. If haemodialysis is not available, a theophylline level more than 100 mg/L should be considered for hemoperfusion. Regular cardiac and seizure monitoring is recommended for individuals taking activated charcoal treatment who have a theophylline level of 20-60 mg/L. Those with a theophylline level exceeding 60 mg/L require close monitoring of the theophylline level, whereas those with levels below 20 mg/L just require a dosage reduction or brief observation [8].

Here, when theophylline toxicity was suspected, the serum theophylline level was promptly checked in order to make a proper diagnosis and determine the best course of treatment. In this case, theophylline toxicity was suspected due to persistent nonspecific gastrointestinal symptoms with sinus tachycardia and a prolonged QT interval against the backdrop of taking regular theophylline without regular theophylline level monitoring. Theophylline was immediately stopped when the serum drug level was found to be at 28.9 ml/L on the third day of the patient’s admission. His theophylline toxicity was managed conservatively in the respiratory ward with continuous cardiac monitoring, regular theophylline level checks, beta blockade, intravenous fluids and electrolyte (potassium and magnesium) replacement. This proved to be the appropriate management of theophylline toxicity in this case, given that his electrolyte imbalance, cardiac arrhythmias, and theophylline level improved over the following days. When theophylline was discontinued, theophylline levels fell rapidly and returned to normal within two days.

## Conclusions

Theophylline is an effective bronchodilator for those with COPD or asthma who do not benefit from beta-2 agonists. Nonetheless, theophylline use has been limited due to its narrow therapeutic window, which necessitates regular theophylline level monitoring, and its potentially life-threatening side effects. Individuals suffering from theophylline toxicity frequently exhibit nonspecific symptoms, resulting in a delay in diagnosis. This case highlights how a lack of regular theophylline monitoring and patient education on theophylline toxicity and side effects resulted in a delay in theophylline toxicity diagnosis. To avoid potentially fatal side effects such as cardiac arrhythmias and seizures, clinicians must have a high suspicion of theophylline toxicity when dealing with those who use theophylline on a regular basis and have nonspecific cardiac or gastrointestinal symptoms.
